# Compliance Study of Endovascular Stent Grafts Incorporated with Polyester and Polyurethane Graft Materials in both Stented and Unstented Zones

**DOI:** 10.3390/ma9080658

**Published:** 2016-08-05

**Authors:** Ying Guan, Lu Wang, Jing Lin, Martin W. King

**Affiliations:** 1 Key Laboratory of Textile Science and Technology, Ministry of Education, College of Textiles, Donghua University, 2999 North Renmin Road, Songjiang, Shanghai 201620, China; ggyf1991@163.com (Y.G.); jlin@dhu.edu.cn (J.L.); martin_king@ncsu.edu (M.W.K.); 2 College of Textiles, North Carolina State University, Raleigh, NC 27695-8301, USA

**Keywords:** stent graft, compliance, stented zone, unstented zone, PU, PET

## Abstract

Compliance mismatch between stent graft and host artery may induce complications and blood flow disorders. However, few studies have been reported on stent graft compliance. This study aims to explore the deformation and compliance of stent graft in stented and unstented zones under three pressure ranges. Compliance of two stent grafts incorporated with polyurethane graft (nitinol-PU) and polyester graft (nitinol-PET) materials respectively were tested; the stents used in the two stent grafts were identical. For the circumferential deformation of the stent grafts under each pressure range, the nitinol-PET stent graft was uniform in both zones. The nitinol-PU stent graft was circumferentially uniform in the stented zone, however, it was nonuniform in the unstented zone. The compliance of the PU graft material was 15 times higher than that of the PET graft. No significant difference in compliance was observed between stented and unstented zones of the nitinol-PET stent graft regardless of the applied pressure range. However, for the nitinol-PU stent graft, compliance of the unstented PU region was approximately twice that of the stented region; thus, compliance along the length of the nitinol-PU stent graft was not constant and different from that of the nitinol-PET stent graft.

## 1. Introduction

The use of endovascular stent grafts for endovascular aneurysm repair (EVAR) has been accepted widely since the first application of an endovascular repair technique to treat an abdominal aortic aneurysm (AAA) reported in 1991 by Parodi [1,2]. However, stent-graft migration, endoleaks [3,4,5,6,7], and fatigue phenomenon [8,9,10,11,12] such as fraying of the yarns and holes in the fabric tubes may occur after surgery, resulting in a high risk of aneurysm rupture [6,13]; secondary interventions are required in approximately 15% to 20% of patients [14].

The change in aneurysm neck diameter (8.4%–13.3%) during cardiac cycle can exceed the maximum diameter of a non-compliant stent graft; thus, stent-graft migration may occur [15,16]. At the same time, compliance mismatch with the host artery can cause biomechanical issues that may trigger local hemodynamic variations including a change in the flow pattern, relative pulsatility, and pulsatile diameter after device implantation [17,18,19,20,21], which could potentially lead to stroke, coronary artery disease, and myocardial infarction [16]. Commercial implant devices also only work for blood transmission and cannot achieve the function of a pulse pump [16].

Some studies have been conducted to investigate the blood flow features in vitro or angiography, hemodynamic, scanning-electron-microscopic, and histological analyses in vivo with compliance matched and compliance mismatched stents. Similarly, finite element analysis was used to evaluate the wall stress and compliance of the stent/artery hybrid structure. The results confirmed that compliance mismatch induced blood flow disturbances; restenosis developed outside the compliance-mismatched stent, whereas compliance-matched stents elicited no or focalized cell accumulations at endings, thereby demonstrating the importance of mechanical properties for stent or other interventional therapies [22,23]. The stent/artery hybrid structure can be compliance matched with proper stent design to reduce stress and hemodynamic disturbance [24]. However, no further details on stent morphology and structure parameters were presented in the aforementioned papers. Nikolaos [25] investigated the effects of different endograft fabric types, such as polyester and polytetrafluoroethylene, on pulse wave velocity (PWV), which is the main parameter of hemodynamics and reflects compliance indirectly. The results indicated that the stent graft incorporated with polyester showed greater PWV and lower compliance. Desai et al. [26] investigated the fatigue resistance of a sutureless aortic stent graft by incorporating a compliant graft made from polyhedral oligomeric silsesquioxane poly(carbonate-urea) urethane (POSS-PCU) nanocomposite polymer with nitinol scaffold. In this study, sutureless technology proved to be a potential method to manufacture stent grafts while also benefiting the development of stent grafts with improved compliance. This paper aims to explore the variation in compliance of an endovascular stent graft, particularly when the stent graft has been assembled with two distinct regions: a stented zone and an unstented zone. The first objective of this study was to show whether the compliance changes along the length of the stent graft between the stented and unstented regions. Since the compliance of stent graft is low and mismatched with the host artery, how to improve the compliance level of the stent graft is a crucial problem. Here, we launched experiments to verify whether a graft’s compliance would make a difference on the compliance of the stent graft, as preliminary fundamental research. Polyurethane (PU), an elastic polymer, is biocompatible [27,28]. It has been widely used as biomaterial, while its degradation and calcification have led to questions of the safety of PU-made biomedical devices [29,30]. PU may initiate stress cracking by the adsorption of lipids [31]. The second objective was to compare the compliance of a polyester (PET) woven fabric that is a less compliant graft with a polyurethane (PU) membrane material that is a more compliant graft when used with the same kind of nitinol stent; the stent grafts were named nitinol-PET and nitinol-PU respectively. The third objective was to establish whether different types of patients (those with hypotensive, normotensive, and hypertensive blood pressures) influence the level of stent-graft compliance in situ. This objective was achieved by measuring the compliance of the stent graft under three different pressure ranges corresponding to the three types of patients.

## 2. Materials and Methods

### 2.1. Grafts

**Figure 1:**
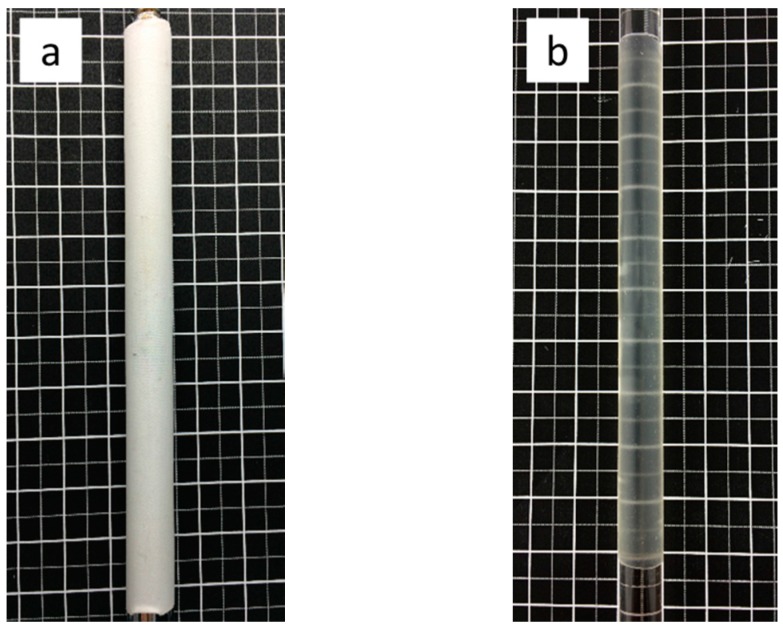
Two grafts: (**a**) polyester (PET) woven graft; (**b**) polyurethane (PU) tubular membrane graft.

The PET woven graft (Figure 1a) was prepared by polyester monofilament yarn (30 D) (Suzhou Suture Needle Company, Suzhou, China) as the warp yarn, while polyester multifilament yarn (20 D/12 f) (Suzhou Suture Needle Company, Suzhou, China) was selected for the weft yarn. Its basic woven structure is plain, wall thickness is 0.117 ± 0.004 mm, warp and weft yarn counts (/cm) are 136.5 ends/cm and 71.9 picks/cm respectively. The structure of the PU graft is the tubular membrane shown in Figure 1b, its thickness is 0.285 ± 0.015 mm. Both of these two grafts have a diameter of 10 mm.

### 2.2. Nitinol Stent

**Figure 2:**
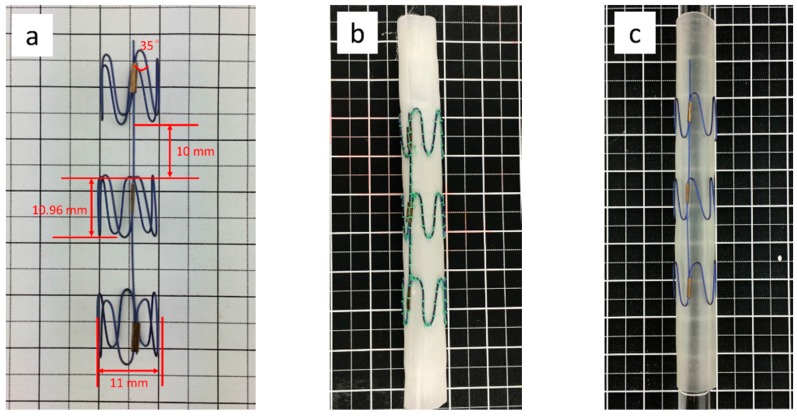
(**a**) Nitinol stent; (**b**) nitinol-PET stent graft; (**c**) nitinol-PU stent graft.

The nitinol stent from Micro-Tech Co. Ltd. (Nanjing, China) consisted of three Z-wires with five apexes, with an angle of 35° for each Z-wire, and a longitudinal bar parallel to the stent-graft axis to provide columnar support. The nitinol filament diameter was 0.4 mm. The structural parameters of the Z-stents are shown in Figure 2a.

### 2.3. Stent Grafts

The two stent grafts shown in Figure 2b,c which incorporated PET and PU grafts, respectively, over the same kind of nitinol stent were assembled at the Biomedical Textile Research Center, Donghua University, Shanghai (China). For the nitinol-PET stent graft, its stent and graft were stitched together with multifilament polyester braided 5-0 suture (Jinhuan Medical Company, Shanghai, China); the PU graft was not sutured with the stent.

### 2.4. Compliance Tests

#### 2.4.1. Test Instruments

**Figure 3:**
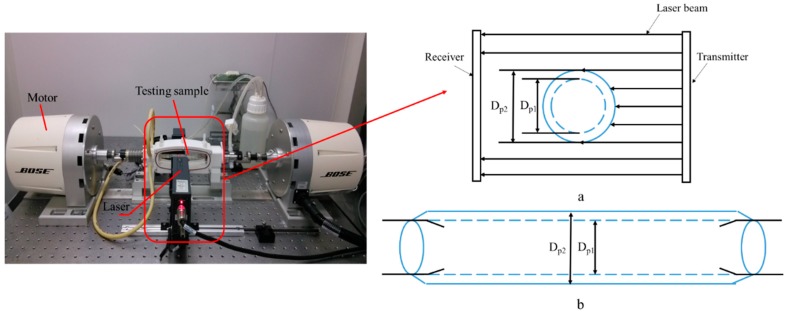
BOSE dynamic simulated system. Schematic of compliance test: (**a**) Cross section; and (**b**) main view, where *D_p_*_1_ (mm) is the diameter under lower pressure and *D_p_*_2_ (mm) is the diameter under higher pressure.

As shown in Figure 3, the compliance tests of the two stent grafts were conducted with a BOSE dynamic simulated system (type 100451, Bose Corporation, Framingham, MA, USA) at a frequency of 1 Hz with three minimum–maximum pulsating pressure ranges from 50 mmHg to 90 mmHg, 80 mmHg to 120 mmHg, and 110 mmHg to 150 mmHg for patients with hypotensive, normotensive, and hypertensive blood pressures, respectively. The circulation liquid in the testing system was distilled water with a flow rate of 100 mL/min. The outer diameter which was measured by the laser beam and pulsing pressure were correspondingly recorded every 0.001 s during the test period.

The compliance (C) calculation formula is shown as follows:

(1) $$C=\frac{\left(S_{p2}-S_{p1}\right)/S_{p1}}{p_{2}-p_{1}}\times 10^{4}$$

where *p*_1_ (mmHg) is the lower pressure value, *p*_2_ (mmHg) is the higher pressure value, *S_p_*_1_ (mm^2^) is the cross-sectional area of lower pressure, and *S_p_*_2_ (mm^2^) is the cross-sectional area of higher pressure.

The outer diameter value along the time of the test was recorded by measuring the sheltered beam width illustrated in Figure 3a,b.

#### 2.4.2. Test Methods

**Figure 4:**
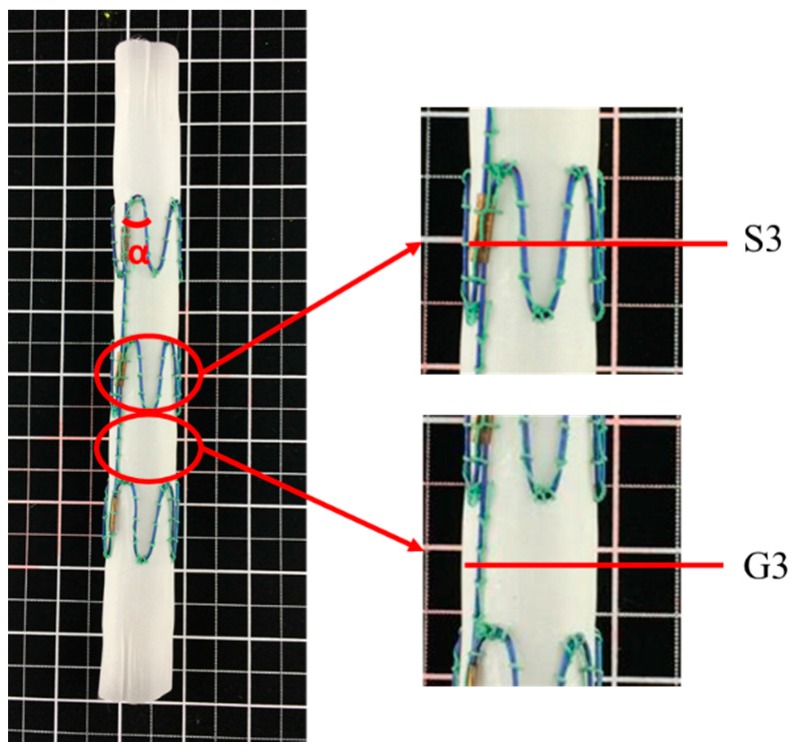
Test sites of stented zone S3 and unstented zone G3 along the longitudinal direction of the Z-stent-supported stent graft.

To fully explore the compliance of the stented and unstented zones of the stent graft, the compliance of two positions along the Z-stent-supported stent-graft axis was tested. The compliance of S3, which was the middle point of the stented zone of the stent graft and representing the compliance of the stented zone, was tested under three pressure ranges. Similarly, the compliance of the middle point of the unstented zone, denoted as G3, representing the compliance of the unstented zone under the three pressure ranges was also tested, as shown in Figure 4.

**Figure 5:**
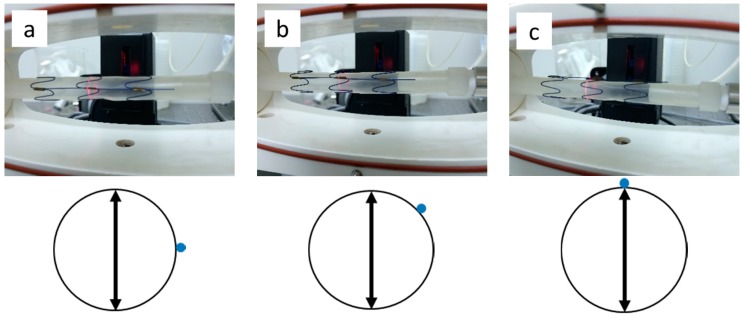
Stent-graft installations when the bar is at the position of (**a**) 0°; (**b**) 45°; and (**c**) 90°. 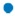 represents the vertical bar.

The stent used in this study was special because it had a longitudinal bar parallel to its axis, which made it similar to the commercial stent graft Talent. The longitudinal bar was designed to provide columnar support and help to stabilize relative position between two Z-wires. The longitudinal bar has been avoided in later generations of stent grafts because it may decrease flexibility of the device; whether it would make a difference on the compliance is still unknown. In this study, both stent grafts were kept straight along their axis when testing. If the compliance property of a device with the longitudinal bar was studied, the compliance property of devices without the longitudinal bar could also be easily understood. However, the test instrument could only measure the diameter change of one position of the sample at a time. To fully explore the circumferential deformation behavior of the stent, the diameter changes of the following three circumferential bar positions were tested separately: when the vertical bar is in front (0°), on top (90°), and between those two positions (45°), as illustrated in Figure 5. For comparison, the diameter changes of both the PET and PU grafts were evaluated under those three circumferential bar positions.

### 2.5. Statistical Analysis

All the data were expressed as mean ± SD. The compliance comparison between and within groups was assessed with one-way ANOVA using SPSS 19 (SPSS Inc., Chicago, IL, USA). A statistically significant difference was considered when *p* was less than 0.05.

## 3. Results and Discussion

### 3.1. Diameter Changes of Both PET and PU Grafts

In order to compare the compliance behavior of both materials, it is useful to relate the compliance behavior to the mechanical properties of both materials. For that purpose, we performed circumferential extension tests on PET tubular graft samples according to the standard method of ISO 7198. The length of the tubular specimen tested was 20 mm. The stretching velocity was 100 mm/min. This test was repeated 3 times. Thus, its modulus and ultimate tensile strength can be calculated.

For the PET graft, there was no significant difference of the diameter changes among the three circumferential positions (0°, 45°, and 90°) regardless of the pressure range. Meanwhile, no significant difference of the diameter changes was detected among the three pressure ranges (50–90 mmHg, 80–120 mmHg, and 110–150 mmHg) regardless of the circumferential positions.

**Figure 6:**
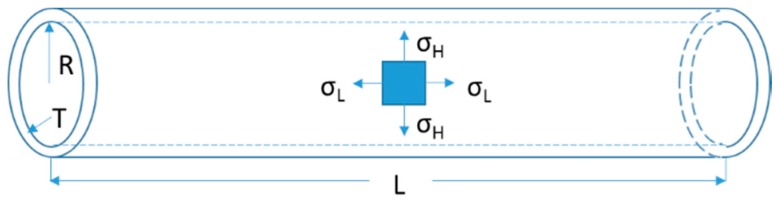
Force decomposition schematic of tubular graft under pressure.

The tubular graft could be regarded as a hollow cylinder when considering its circumferential force. If the inner pressure *P* was applied, according to the force analysis of the graft wall, it can be decomposed into circumferential stress $\sigma_{H}$ and longitudinal stress $\sigma_{L}$, shown in Figure 6. According to the force balance, the equation between inner pressure and circumferential wall stress was given by

(2) $$P\times \left(2\times R\times L\right)=\sigma_{H}\times \left(2\times T\times L\right)$$

where *P* is the inner pressure (MPa), *R* is the radius of the tubular graft (mm), *L* is the length of the tubular graft (mm), and *T* is the wall thickness of the tubular graft (mm).

Circumferential stresses $\sigma_{H}$ under those individual pressures could be calculated correspondingly via Equation (1), as shown in Table 1. *R* is 5 mm for PET graft, and *T* is 0.127 mm.

**Figure 7:**
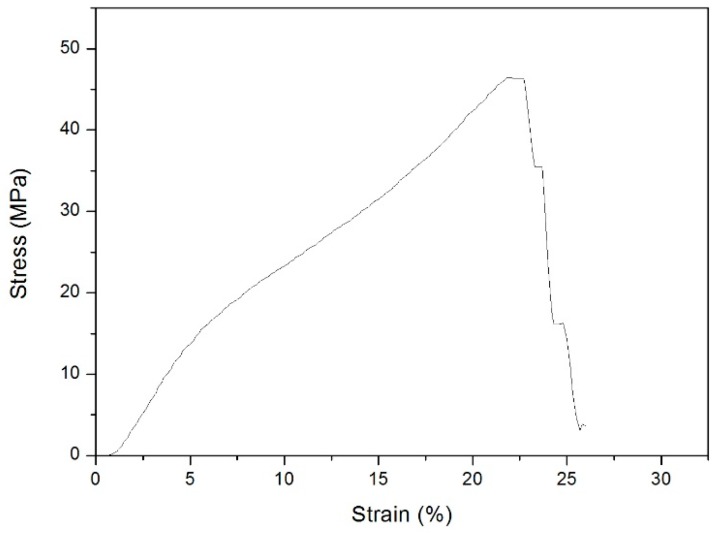
Circumferential tensile stress-strain curve of the PET tubular graft.

PET graft’s circumferential tensile strength was tested; its tensile strain under each stress value was obtained as well as the modulus of the graft. A representation of the PET tubular graft stress-strain curve is shown in Figure 7. The fracture stress of the PET graft calculated was 44.1 MPa. The modulus of the PET tubular graft was 312.7 MPa.

**Figure 8:**
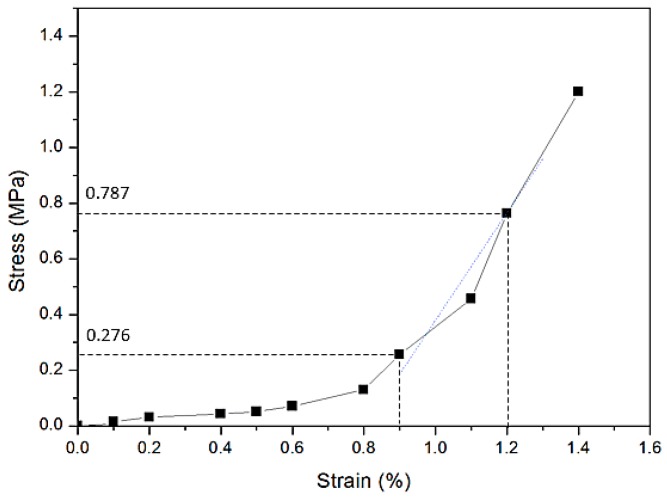
Initial part of the circumferential tensile stress-strain curve of the PET tubular graft that contains the stress range corresponding to compliance test pressures and the fitting line of these compliance test pressure ranges.

However, the circumferential stresses imposed on the PET graft were low when compared with PET graft’s ultimate tensile stress, so the particular stress-strain curve under low stress is depicted in Figure 8. From the stress-strain curve, we can see that the PET graft had tiny deformations circumferentially under each pressure range. The gentle slope in the early stage of Figure 8 may represent filament rearrangement within the woven fabric, and then the PET graft showed an abrupt slope which indicates that the PET graft may have experienced an elongation of the yarns under the following increased load. The stresses imposed on the graft (stress from 0.276 to 0.787 MPa) all fall into the abrupt slope, meanwhile, the PET graft displayed as an elastic phase fitted with a dashed straight line whose slope, also called modulus, is 177.2 MPa, illustrated in Figure 8. As the strain difference values of the PET graft under three pressure ranges are the same, so the diameter change under those three pressure ranges does not show a significant difference.

**Figure 9:**
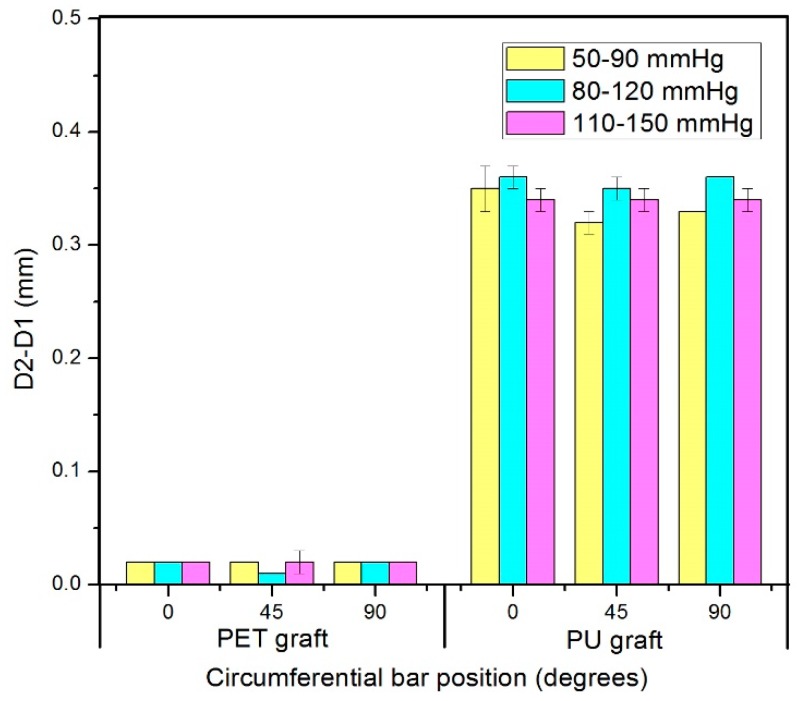
Diameter changes of PET and PU grafts in three circumferential bar positions.

The diameter changes of the PU graft showed no significant differences among the three circumferential bar positions (0°, 45° and 90°) under each pressure range (Figure 9). However, its diameter changes showed a significant difference between 50–90 mmHg and 110–150 mmHg at the 45° position and between 50–90 mmHg and 80–120 mmHg at the 90° position.

As shown in Figure 9, the diameter change of the PU graft was significantly higher than that of the PET graft under each pressure range and circumferential position because PET has a significantly higher modulus than PU [32]. This result can be explained by their chain structures. PET chain, which is highly symmetrical, consists of three components: soft segment, a rigid benzene ring, and a polar ester group. The presence of the benzene ring in the molecular chain give them a rigid structure. The polymer chains parallel with each other in one orderly arrangement. They make PET fibers resistant to deformation. PU are composed of two types of segments: one long and amorphous segment (soft segment) giving the polymer elasticity, the other a short and rigid segment (rigid segment) giving the polymer strength. Those two types of segments repeatedly arranged is benefit to polymer’s elasticity. The ratio between the diameter change of the PU and PET grafts was calculated. The diameter change of the PU graft was at least 15 times higher than that of the PET graft.

For both the PET and PU grafts, the diameter change showed no significant difference among the circumferential positions under a certain pressure range due to their homogeneous wall structure, indicating that the grafts experienced uniform deformation circumferentially. The shape of the cross section of the grafts remained circular under each pressure range.

### 3.2. Compliance of Both PET and PU Grafts

**Figure 10:**
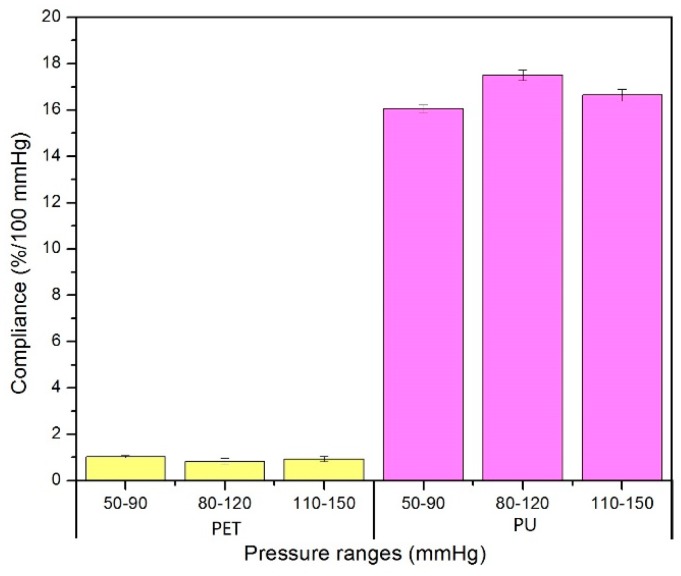
Compliance of PET and PU grafts under three pressure ranges.

**Figure 11:**
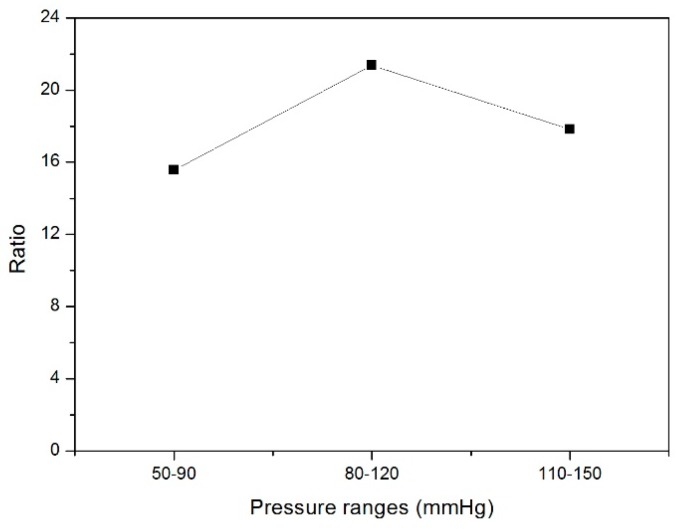
Ratio of compliance between PU and PET grafts.

The cross-section area change under particular pressure ranges can be calculated according to its diameter, as well as its compliance, as shown in Figure 10. The diameter change of the PU graft is at least 15 times higher than that of the PET graft, and so are their compliances (Figure 11); meanwhile, the data showed the same trend. Since compliance is calculated via the equation with the parameters of one particular pressure range and its corresponding cross-section area change, it is reasonable that compliance results and diameter change showed the same trend under three continuous pressure ranges. The compliance of the PET graft showed no significant difference among the three pressure ranges while the compliance of the PU graft under 80–120 mmHg showed higher compliance than that under the other two pressure ranges.

### 3.3. Diameter Changes of Nitinol-PET and Nitinol-PU Stent Grafts

**Figure 12:**
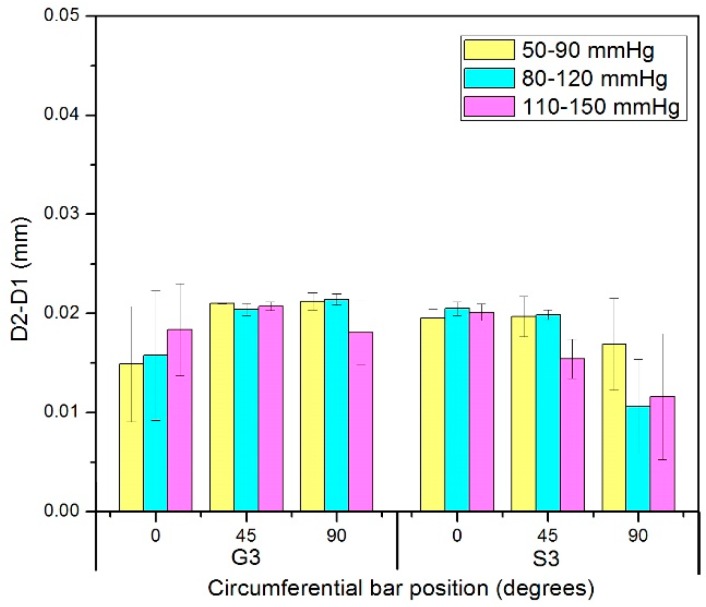
Diameter changes of stented zone S3 and unstented zone G3 of the nitinol-PET stent graft in three circumferential bar positions.

The diameter change of both stent grafts were evaluated in the middle point of stented zone S3 and unstented zone G3, as shown in Figure 4. The result of diameter change in the nitinol-PET stent graft (Figure 12) showed no significant difference among the three circumferential bar positions (0°, 45°, and 90°) under each pressure range in stented zone S3 and unstented zone G3, indicating that the cross-section shape of both stented zone S3 and unstented zone G3 in the nitinol-PET stent graft remained circular under the three pressure ranges, which was consistent with that of the PET graft alone shown in Figure 9. The diameter changes of stented zone S3 and unstented zone G3 showed no significant difference; thus, the existence of nitinol stent in the nitinol-PET stent graft does not cause a difference in its diameter change between stented zone S3 and unstented zone G3. The result of the diameter change of the nitinol-PET stent graft showed no significant difference with increased pressure ranges whether in stented zone S3 or unstented zone G3, which was consistent with the result of the PET graft.

For the nitinol-PU stent graft shown in Table 2, no significant differences existed in stented zone S3 among three circumferential bar positions under each pressure range, indicating that its cross-section shape remained circular. However, in the unstented zone G3, the diameter change in the circumferential bar position at 90° was significantly lower than that at 0° and 45° (*p* value was 0.014 and 0.021 under 50–90 mmHg, 0.026 and 0.043 under 80–120 mmHg, 0.044 and 0.031 under 110–150 mmHg, respectively). Meanwhile, no significant difference in diameter change was detected between the circumferential bar positions at 0° and 45° (*p* value was 0.989, 0.983 and 0.964 under three the pressure ranges respectively). The existence of the vertical bar should be the main factor that caused the non-uniform diameter change circumferentially in the unstented zone of the nitinol-PU stent graft which greatly inhibited the graft’s diameter expansion compared with the diameter change results of the PU graft alone. When the circumferential bar position was at 90°, the diameter change value under the pressure range of 50–90 mmHg showed a significantly lower result than that of the pressure ranges of 80–120 mmHg and 110–150 mmHg, respectively, in both stented zone S3 (*p* value was 0.029 and 0.018 separately) and unstented zone G3 (*p* value was 0.018 and 0.033 separately). No other significant difference in diameter change among the three pressure ranges was observed except in the circumferential bar position at 0° in the stented zone S3 between 80–120 mmHg and 110–150 mmHg (*p* value was 0.029).

**Figure 13:**
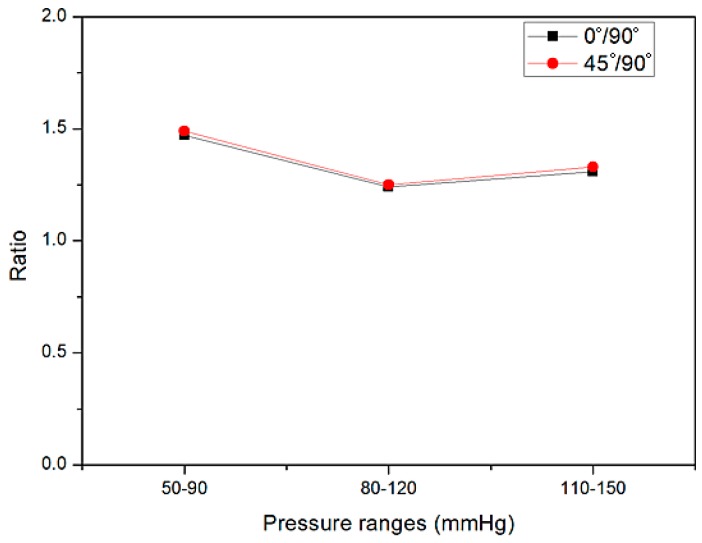
Ratios of diameter change in unstented zone G3 of the nitinol-PU stent graft between circumferential bar position at 0° and 90° and at 45° and 90°.

The diameter change ratios between 0° and 90° and between 45° and 90° were calculated and graphed in Figure 13. The diameter change values of both 0° and 45° positions were at least 1.2 times higher compared with that of the position at 90° under the pressure range of 80–120 mmHg and reached 1.5 times under the pressure range of 50–90 mmHg. Thus, the cross-section shape of the unstented PU was non-circular and similar to being “heart-shaped”, which was significantly different from the circular cross-sectional shape observed in the PU-stented region. There is only a vertical bar in the unstented zone G3; the restriction was imposed on the graft membrane under the vertical bar, since the nitinol stent was more rigid compared with the PU graft, while the graft membrane, except where it was under the vertical bar, can be expanded under the pressure. The condition in the stented zone S3 is quite different; it is covered by a rigid stent with five apexes whose arch angle is 35°, thus the expansion of the tubular graft in the stented region S3 is greatly restricted compared with that of unstented region G3.

**Figure 14:**
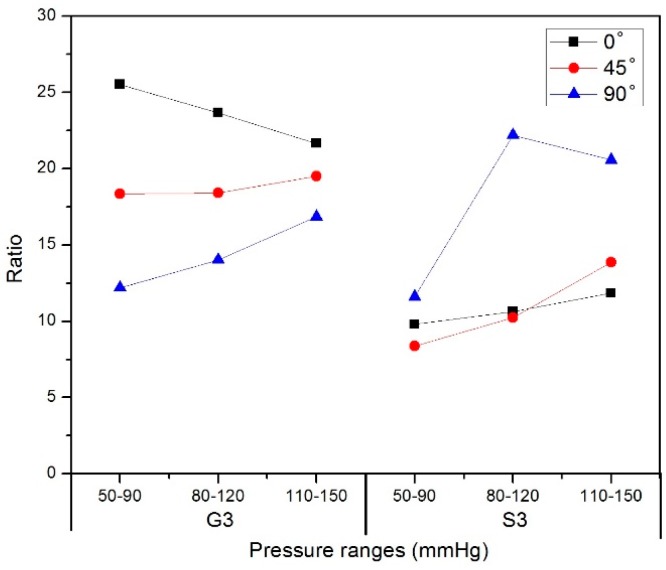
The ratio of diameter change between nitinol-PU and nitinol-PET stent grafts in unstented zone G3 and stented zone S3.

The diameter change in the nitinol-PU stent graft was at least 8 times higher than that in the nitinol-PET stent graft in the stented zone S3 and 12 times higher than that in the unstented zone G3 (Figure 14), which shows the same trend of the PU graft compared with the PET graft. Both nitinol-PET and nitinol-PU are incorporated with the same kind of nitinol stent, while their diameter change under the same pressure range is quite different. It shows that the tubular graft property makes a big difference in the diameter expansion of stent grafts; a more compliant tubular graft would be helpful to enhance the diameter change of a stent graft. The ratio between diameter change of both the PET and PU tubular graft were at least 15 times.

For the stented zone of the nitinol-PU stent graft, its diameter changed with the pressure wave so stent deformation must have occurred simultaneously. A possible deformation pattern is the apex angle changing rather than the nitinol stent filament elongating due to its high modulus (when the diameter of nitinol filament is 0.2 mm, its modulus reaches 31.36 GPa, when the diameter of nitinol filament is 0.5 mm its modulus is 30.32 GPa). The nitinol stent consisted of three Z-wires with five apexes whose initial angle (α) with no internal pressure was 35° for each Z-wire. The apex angle α could be increased slightly with increased pressure. According to the diameter of the stent graft, each apex angle can be calculated; it increased to 37.47° at 150 mmHg, and the increase amounts were 0.59°, 0.72°, and 0.76° under pressure ranges of 50–90 mmHg, 80–120 mmHg, and 110–150 mmHg, respectively.

### 3.4. Compliance of Nitinol-PET and Nitinol-PU Stent Grafts

**Figure 15:**
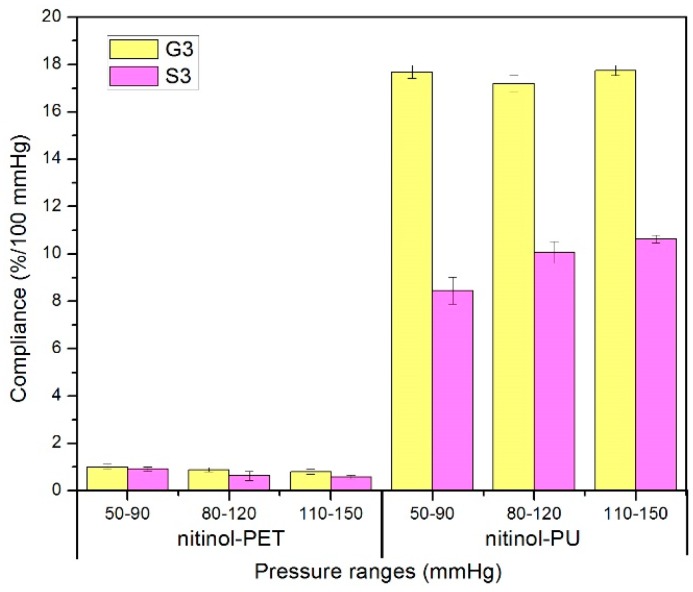
Compliance of nitinol-PET and nitinol-PU stent grafts in unstented zone G3 and stented zone S3.

The compliance of the nitinol-PET and nitinol-PU stent grafts are shown in Figure 15. For the nitinol-PET stent graft, the compliance under the pressure range of 50–90 mmHg is significantly higher than that under the range of 110–150 mmHg in stented zone S3, whereas no significant difference among the three pressure ranges was observed in the unstented zone. If the nitinol-PET stent graft was used on a hypertensive patient, it would show the lowest compliance result. Moreover, no significant difference existed between the stented zone S3 and the unstented zone G3 under the same pressure range. For the nitinol-PU stent graft, a significant difference in compliance was observed between the pressure ranges of 50–90 mmHg and 80–120 mmHg and between the pressure ranges of 50–90 mmHg and 110–150 mmHg at the same time in stented zone S3. This result indicates that the nitinol-PU stent graft will show higher compliance in normotensive and hypertensive patients than hypotensive ones. No significant difference was found in the unstented zone of the nitinol-PU stent graft under different pressure ranges. If the compliance becomes a parameter taken into consideration in the near future when doctors choose the optimum stent graft, then the blood pressure condition of the patient should be a key factor since it will make a difference on the compliance property.

**Figure 16:**
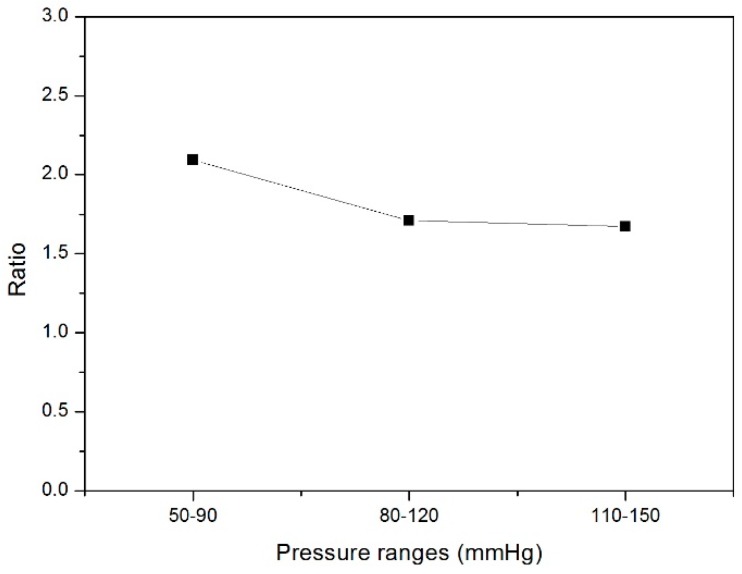
Ratio of compliance between G3 and S3 of nitinol-PU stent graft.

**Figure 17:**
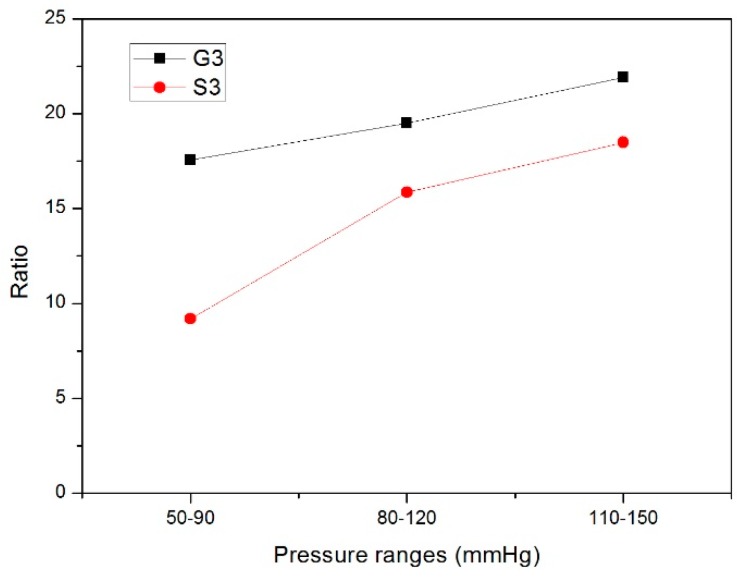
Ratio of compliance between nitinol-PU stent graft and nitinol-PET stent graft in stented zone S3 and unstented zone G3.

The radial deformation in the stented zone of the nitinol-PU stent graft was limited by the stent. Along the length of the stent graft, the amount of compliance of the unstented zone G3 was approximately twice that of the stented region S3 (Figure 16); thus, incorporating the nitinol stent caused the compliance to be non-uniform longitudinally. The compliance of the nitinol-PU stent graft was higher than that of the nitinol-PET stent graft by at least 17 times in the unstented region G3 and 9 times in the stented region S3 (Figure 17). The results confirmed that stent-graft compliance was extremely relevant with graft elasticity. Overall, a more compliant tubular graft could improve the compliance of the stent graft as a whole both in stented zone S3 and unstented zone G3.

A less compliant stent graft could cause biomechanical issues, mainly changes in the flow pattern, relative pulsatility, and pulsatile diameter after device implantation. This kind of complication may be remitted by introducing a stent graft incorporated with a more compliant tubular graft than the PET graft. However, the compliance of the stent graft with a more compliant graft are not uniform longitudinally. There is a big difference between stented zone S3 and unstented zone G3 in our research; this could lead to new blood flow disturbances. In this study, only one kind of stent structure was studied. Different stent structural parameters, such as apex angle, apex number circumferentially, and the length of stent region, may correspond to different radical deformation characteristics and compliance properties. More types of stents with various structures need to be evaluated in a further study to determine the superior stent structure which makes the stent graft have uniform compliance between stented zone S3 and unstented zone G3 longitudinally.

## 4. Conclusions

In this study, the variation in compliance was measured along the length of stent grafts with distinct stented and unstented regions. Stent grafts incorporating either PET or PU graft materials were included.

(1) The PU graft material showed significantly greater compliance than the PET graft material. The difference was at least 17 times higher.
(2) Analysis of the nitinol-PET stent graft showed no difference in the amount of compliance between the stented and unstented zones regardless of the applied pressure range.
(3) For the nitinol-PU stent graft, significant differences were observed in the amount of compliance in the stented and unstented regions. Thus, the compliance of the unstented PU region was approximately twice that of the stented region along the length of the stent graft. In addition, the pressure range was observed to influence the compliance of the stented region, with hypertensive pressures being associated with the highest compliance values in the stented zone S3 of the nitinol-PU stent graft.
(4) Furthermore, because the stent graft was constructed with a longitudinal bar connecting the stents, the nitinol-PU stent graft was associated with a different kind of non-uniformity along its length, different from that of nitinol-PET stent graft. The shape of the unstented PU cross-section was found to be non-circular and rather “heart-shaped”, which was significantly different from the circular cross-sectional shape observed in the PU-stented region. Further work needs to be undertaken so as to translate these cross-sectional size and shape changes into disruptions of laminar flow through the stent graft.

## Figures and Tables

**Table 1:** $\sigma_{H}$ of PET graft under each inner pressure.

Pressure/mmHg	50	90	80	120	110	150
$\sigma_{H}$/MPa	0.276	0.472	0.421	0.630	0.579	0.787

**Table 2:** Diameter changes in stented zone S3 and unstented zone G3 of the nitinol-PU stent graft under three circumferential bar positions (mm).

Test Positions	Bar Positions	Pressure Range/mmHg		
		50–90	80–120	110–150
G3	0°	0.380 ± 0.023	0.373 ± 0.024	0.398 ± 0.034
	45°	0.385 ± 0.031	0.376 ± 0.023	0.404 ± 0.029
	90°	0.258 ± 0.007	0.301 ± 0.012	0.304 ± 0.013
S3	0°	0.192 ± 0.025	0.218 ± 0.009	0.238 ± 0.003
	45°	0.165 ± 0.019	0.204 ± 0.026	0.214 ± 0.021
	90°	0.196 ± 0.012	0.235 ± 0.013	0.239 ± 0.013
